# Supplementary material for: Social learning strategies modify the effect of network structure on group performance
Source: Nat Commun. 2016 Oct 7;7:13109. doi: 10.1038/ncomms13109 (PMC5059778; doi:10.1038/ncomms13109)
Supplement: Supplementary Information — Supplementary Figures 1-5, Supplementary Tables 1-3, Supplementary Notes 1-4, Supplementary References [file ncomms13109-s1.pdf]

Supplementary Figures

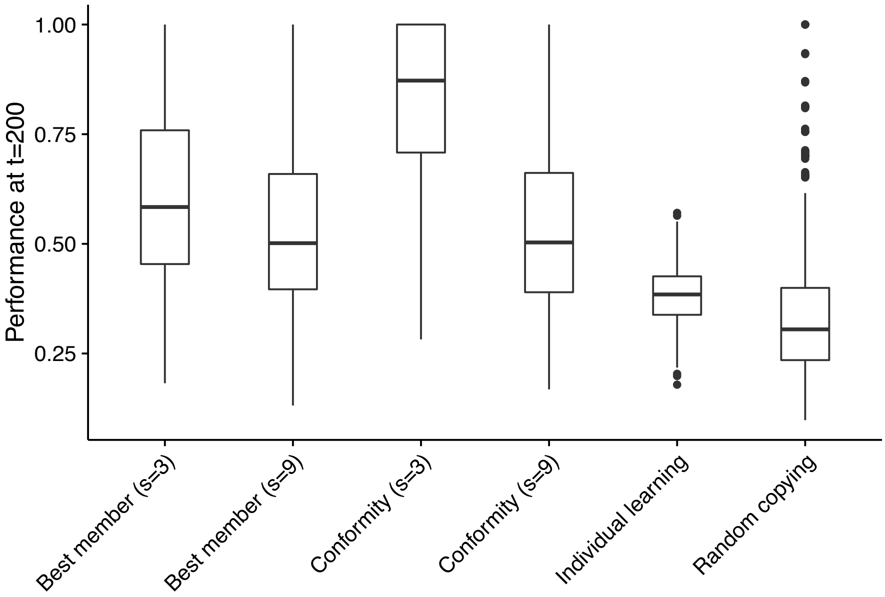

Supplementary Figure1: Boxplot showing performance variability across repetitions for each social learning strategy. The lower and upper "hinges" correspond to the first and third quartiles (the 25th and 75th percentiles), the upper (lower) whiskers extend from the hinge to the highest (lowest) value that is within 1.5 * *IQR* (inter-quartile range). Data beyond the end of the whiskers are outliers and are plotted as points.

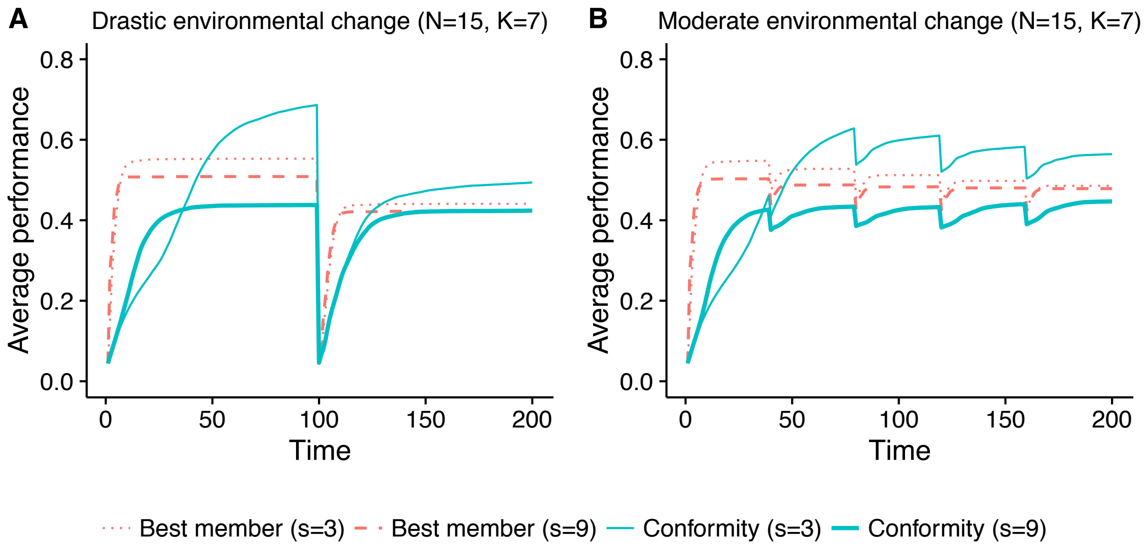

Supplementary Figure 2: Performance over time for different strategies in changing environments. (**a**) Rare but drastic environmental change. (**b**) Frequent but less drastic environmental change. Environmental change does not alter the main pattern of results.. Red dotted lines: best member (s=3); red dashed lines: best member (s=9); turquoise thin lines: conformity (s=3); turquoise thick lines: conformity (s=9).

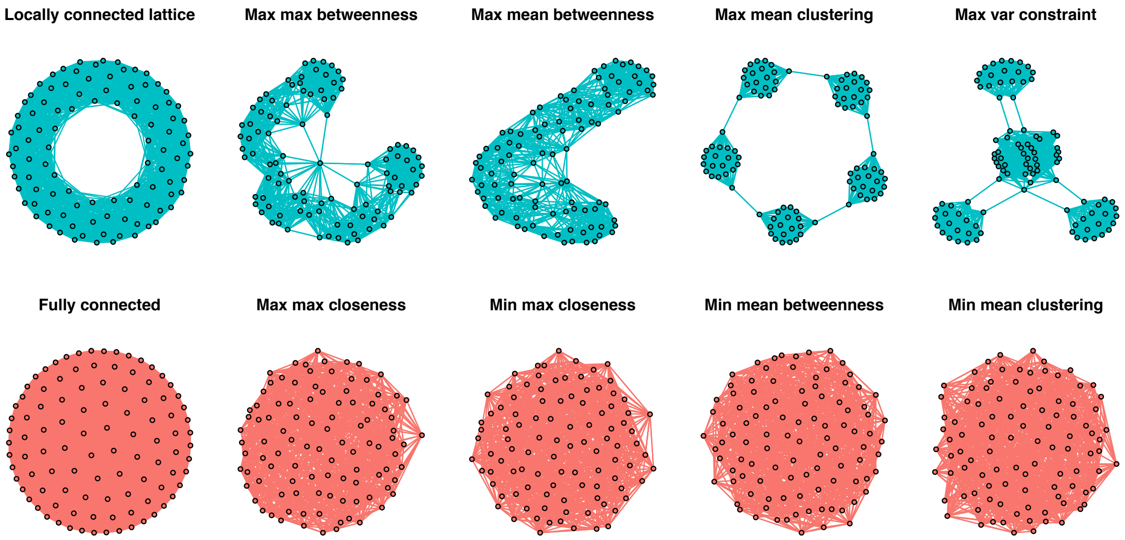

Supplementary Figure 3: Plots of the networks studied. Top panel shows 'inefficient' networks, bottom panel shows 'efficient' networks.

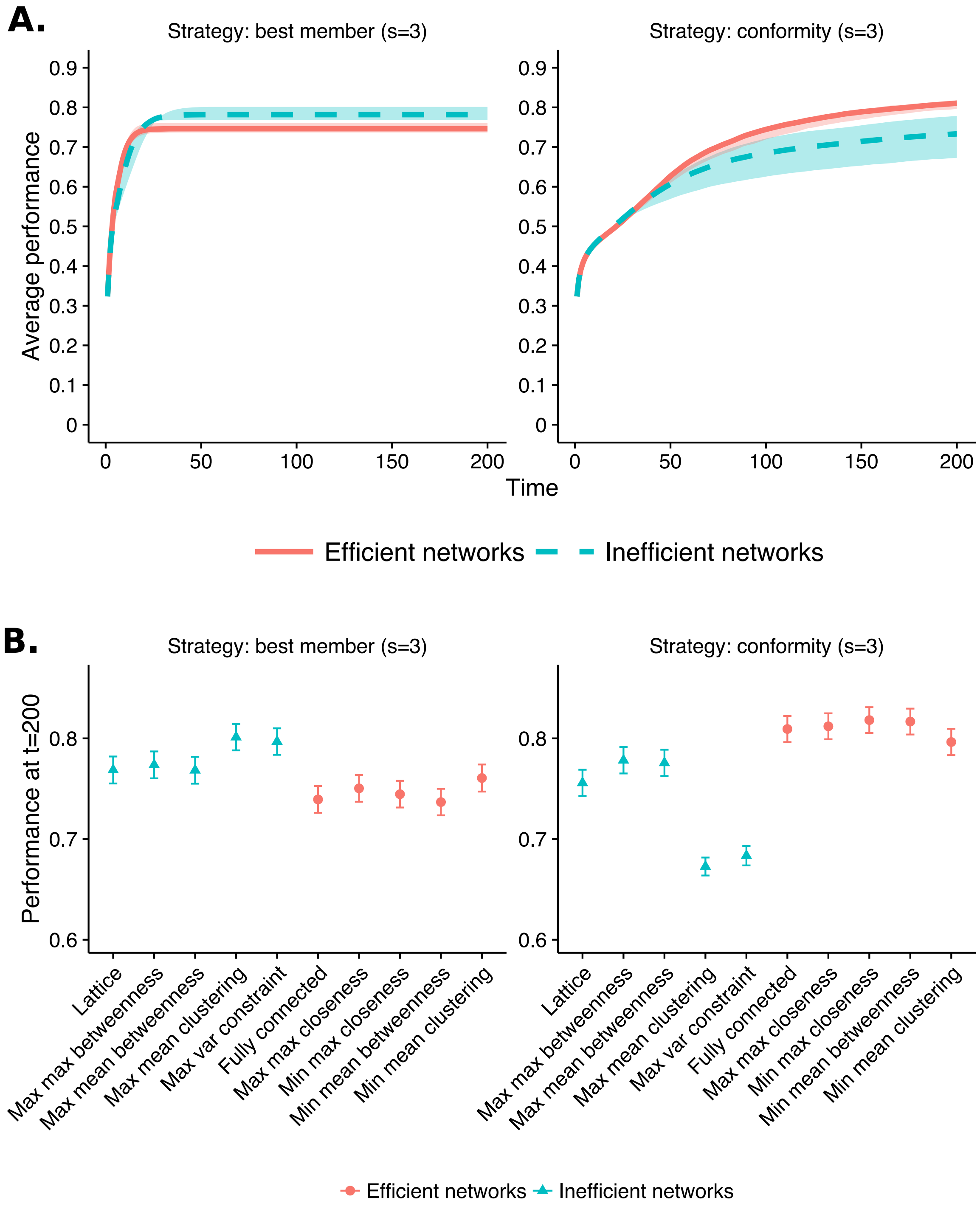

Supplementary Figure 4: Performance of different networks as a function of social learning strategy assuming a 2D landscape (see Supplementary Note 2). A: Group performance averaged across 'efficient' (red solid lines) and 'inefficient' (turquoise dashed lines) networks. Shadings around the lines show the region between the best and worst performing network in each category. B: Average performance at the final time step (*t = 200*) for each network. Error bars show +- 2 standard error of the mean. Left panels:

individuals rely on the ‘best member’ (s = 3) strategy; Right panels: individuals rely on the ‘conformity’ (s = 3) strategy. ‘Inefficient’ networks outperform ‘efficient’ networks when individuals rely on the ‘best member’ strategy. ‘Efficient networks’ outperform ‘inefficient’ networks when individuals rely on the ‘conformity’ strategy. Based on *N=100* agents and 1000 repetitions.

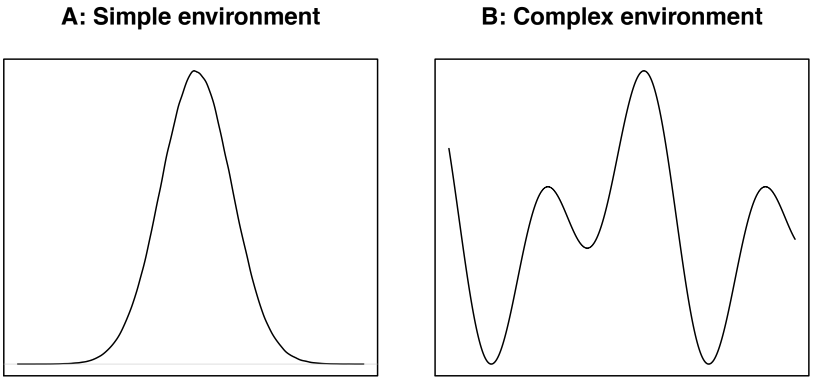

Supplementary Figure 5: Simplified illustration of the two task environments studied. (**a**) Simple environment with a single global optimum. (**b**) Complex environment with multiple local optima and a global optimum. In the simple environment nearby solutions have very similar payoffs; therefore, gradual improvement of a solution will eventually lead to the global optimum. In the complex environment payoffs of nearby solutions can be very different, therefore, gradual improvement of a solution can lead to local optima from which it is impossible to improve and, as a result, to find the global optimum.

Supplementary Tables

Supplementary Table 1: Strategy performance at the final time step (t=200) in different environments. We explore landscapes with *N=15* and *K*=[2,4,6,7,8,10,12,14]. Our choices for values of N and K are representative of the literature. We report the mean performance of each strategy, maximum and minimum performance and standard deviation.

Strategy		Environment						
		N=15, *K*=2	*N*=15, *K*=4	*N*=15, *K*=6	*N*=15, *K*=8	*N*=15, *K*=10	*N*=15, *K*=12	*N*=15, *K*=14
best member (*s*=3)	mean	0.91	0.79	0.66	0.57	0.49	0.45	0.40
	min	0.35	0.22	0.18	0.17	0.12	0.14	0.15
	max	1	1	1	1	1	1	1
	st. dev	0.13	0.19	0.21	0.19	0.19	0.17	0.15
best member (*s*=9)	mean	0.87	0.73	0.60	0.51	0.44	0.39	0.37
	min	0.22	0.19	0.16	0.17	0.13	0.10	0.11
	max	1	1	1	1	1	1	1
	st. dev	0.16	0.22	0.21	0.20	0.19	0.15	0.15
conformity (*s*=3)	mean	0.99	0.96	0.87	0.75	0.61	0.50	0.40
	min	0.65	0.55	0.32	0.24	0.18	0.14	0.13
	max	1	1	1	1	1	1	1
	st. dev	0.03	0.08	0.15	0.19	0.19	0.18	0.15
conformity (*s*=9)	mean	0.86	0.72	0.59	0.50	0.43	0.57	0.32
	min	0.39	0.24	0.20	0.15	0.10	0.09	0.11
	max	1	1	1	1	1	1	1
	st. dev	0.16	0.21	0.21	0.19	0.17	0.15	0.13
random copying	mean	0.45	0.37	0.34	0.33	0.33	0.33	0.33
	min	0.09	0.11	0.08	0.07	0.09	0.07	0.10
	max	1	1	1	1	1	1	1
	st. dev	0.16	0.15	0.14	0.14	0.14	0.14	0.15
individual learning	mean	0.68	0.52	0.42	0.35	0.30	0.26	0.23
	min	0.44	0.29	0.18	0.17	0.12	0.10	0.09
	max	0.95	0.73	0.61	0.53	0.46	0.4	0.36
	st. dev	0.09	0.07	0.07	0.06	0.05	0.05	0.04

Supplementary Table 2: Strategy performance at the final time step (*t*=200) as a function of sample size. Note that the conformity strategy requires at least a sample of *s*=3 in order to assess the most frequent option.

	s=1	*s*=2	*s*=3	*s*=5	*s*=7	*s*=9	*s*=19
best member	0.72	0.64	0.62	0.59	0.57	0.55	0.52
conformity	N/A	N/A	0.82	0.72	0.62	0.55	0.45

Supplementary Table 3: Properties of the networks studied. The top five networks are efficient, while the bottom five networks are inefficient, as indicated in particular by their diameter and clustering coefficients.

Topology	Radius	Diameter	Closeness	Betweenness	Clustering	Constraint
Efficient networks						
Fully connected	1	1	1	0	1	0.04
Max max closeness	2	3	0.55	0.01	0.17	0.07
Min max closeness	2	3	0.55	0.01	0.18	0.07
Min mean betweenness	2	2	0.55	0.01	0.18	0.07
Min mean clustering	2	3	0.55	0.01	0.01	0.05
Inefficient networks						
Max max betweenness	2	4	0.37	0.02	0.73	0.15
Max mean clustering	7	7	0.26	0.03	0.98	0.20
Max mean betweenness	3	6	0.34	0.02	0.86	0.14
Max var constraint	3	5	0.35	0.02	0.59	0.14
Locally connected lattice	3	6	0.32	0.02	0.73	0.08

Supplementary Notes

Supplementary Note 1

Best sample size. We focus on identifying the best sample size for the best member and conformity strategies, assuming a fully connected network. Supplementary Table 2 shows performance of the main strategies at the final time step (t=200) as a function of sample size. For the best member strategy the best sample size turns out to be s=1, however, since it does not change any of our main conclusions, we chose to keep sample size of s=3 in the main text to make it directly comparable to the conformity strategy that required a minimum sample size of s=3 (note also that s=3 is the sample size that participants in Mason and Watts[1] had access to). For conformity the best sample size is s=3 and increasing sample size above 3 quickly decreases performance.

Supplementary Note 2

Changing task environments. We model two cases of environmental change. In the first case we completely regenerate the NK landscape (N=15, K=7) half-way through the simulation, forcing individuals to re-learn everything that was adaptive in the past. This represents an environment that changes rarely but drastically (for an example see ref. [1]). In the second case we redraw the fitness contribution of a randomly selected single digit in the solution space every 40th time step. This represents a more frequent, but less drastic change. For simplicity, we assume a fully connected network. From Supplementary Figure 2 we conclude that environmental change does not alter our main conclusions regarding strategy performance. Conformity with small samples remains the best strategy, followed by best member with small samples.

Supplementary Note 3

Properties of the networks studied. Supplementary Table 3 shows the properties of all the networks we studied. In addition to a 'fully connected network' where each individual is connected to everyone else in the population and a 'locally connected lattice' where individuals are connected to their d immediate neighbors, we included eight network structures that were proposed in a recent study focusing on the relationship between network structure and group performance[2] (see Supplementary Figure 3). Each of these eight networks were constructed to maximize or minimize a specific network measure, namely: (a) closeness centrality, or the average length of shortest paths between a node and all other nodes, (b) betweenness centrality, or the average proportion of shortest paths between pairs of nodes that pass through a node, (c) clustering coefficient, or the average connectedness of a node's neighbors, and (d) network constraint, or the average extent to which a node bridges different groups of individuals (see column 'Topology' in Supplementary Table 3). In addition, we measured the diameter of the network, that is, the shortest distance between the two most distant nodes in the network. The exact method for constructing the networks is reported in the Methods section.

The top five networks in the table are efficient at spreading information, while the bottom five networks are inefficient, as indicated by their diameter and clustering

coefficients (for both measures, higher values typically indicate less efficiency, with the exception of the fully connected network in which the whole network is composed of a single cluster). Note that the classification of two networks (Min max closeness (efficient) and Max var constraint (inefficient)) differ from the classification in ref. [2]. This is because in our larger node networks lead to different network measures for these networks, leading to a different classification.

Supplementary Note 4

Performance on the Mason and Watts landscape. In our analyses we focused on the *NK* fitness landscape, which is an *N*-dimensional environment. However, the study of Mason and Watts[1] focused on a 2-dimensional landscape. To see whether our main argument also applies to the landscape they studied, we re-run our main analyses on this 2-dimensional landscape. We use the Supporting Information provided in Mason and Watts[1] to construct the landscape and provide the code (see Code availability section in the main text).

All details remain the same as in our main study, except for how individual learning is performed. Mason and Watts[1] try a number of different individual learning strategies in their attempt to reconstruct the inefficient network results. They find mostly no difference between networks in their simulations (see pages 11-13 in their Supplementary Materials). Note, however, that they only ran their studies for 15 time steps, which we believe is insufficient to assess the long-run performance of networks. Here we propose an individual learning rule that does replicate the finding that inefficient network perform better when individuals rely on the 'best member' *(s=3)* strategy, while efficient networks perform better when individuals rely on 'conformity' *(s=3).* Following Mason and Watts[1] we assume that individuals are myopic in their search and are only able to evaluate solutions within their search radius (defined as the distance of search from their current solution, see also page 3 in the Supporting Information of Mason and Watts[1]). We assume that on each trial individual's search radius is a number sampled uniformly between 1 and the length of the grid (100).

Supplementary Figure 4 show the results. We are able to replicate our main finding that 'efficient' networks outperform 'inefficient' networks when individuals rely on 'conformity' *(s=3)* while 'inefficient' networks are better than 'efficient' networks when individuals rely on the 'best member' *(s=3)* strategy. Overall, these results indicate that the contradictory results in the literature might result from the social learning strategies used by individuals.

Supplementary References

1. Mason, W. A., & Watts, D. J. Collaborative learning in networks. *Proceedings of the National Academy of Sciences* **109,** 764-769 (2012).

2. Whitehead, H. & Richerson, P. J. The evolution of conformist social learning can cause population collapse in realistically variable environments. *Evolution and Human Behavior* **30,** 261-273 (2009.
